# Biological Characteristics and Predictive Model of Biopsy-Proven Acute Rejection (BPAR) After Kidney Transplantation: Evidences of Multi-Omics Analysis

**DOI:** 10.3389/fgene.2022.844709

**Published:** 2022-03-21

**Authors:** Qianguang Han, Xiang Zhang, Xiaohan Ren, Zhou Hang, Yu Yin, Zijie Wang, Hao Chen, Li Sun, Jun Tao, Zhijian Han, Ruoyun Tan, Min Gu, Xiaobing Ju

**Affiliations:** ^1^ Department of Urology, The First Affiliated Hospital of Nanjing Medical University, Nanjing, China; ^2^ Department of Urology, Affiliated Hospital of Nantong University, Nantong, China; ^3^ Department of Urology, The Second Affiliated Hospital of Nanjing Medical University, Nanjing, China

**Keywords:** biopsy-proven acute rejection (BPAR), kidney transplantation, bioinformatics analysis, predictive model, gene expression omnibus

## Abstract

**Objectives:** Early diagnosis and detection of acute rejection following kidney transplantation are of great significance for guiding the treatment and improving the prognosis of renal transplant recipients. In this study, we are aimed to explore the biological characteristics of biopsy-proven acute rejection (BPAR) and establish a predictive model.

**Methods:** Gene expression matrix of the renal allograft samples in the GEO database were screened and included, using Limma R package to identify differentially expressed transcripts between BPAR and No-BPAR groups. Then a predictive model of BPAR was established based on logistic regression of which key transcripts involved in the predictive model were further explored using functional enrichment analyses including Gene Ontology analysis (GO), Kyoto Encyclopedia of Genes and Genomes (KEGG) pathway analysis, and Gene Set Enrichment Analysis (GSEA).

**Results:** A total of four studies (GSE129166, GSE48581, GSE36059, and GSE98320) were included for extensive analysis of differential expression. 32 differential expressed transcripts were observed to be significant between two groups after the pooled analysis. Afterward, a predictive model containing the five most significant transcripts (IDO1, CXCL10, IFNG, GBP1, PMAIP1) showed good predictive efficacy for BPAR after kidney transplantation (AUC = 0.919, 95%CI = 0.902–0.939). Results of functional enrichment analysis showed that The functions of differential genes are mainly manifested in chemokine receptor binding, chemokine activity, G protein-coupled receptor binding, etc. while the immune infiltration analysis indicated that immune cells mainly related to acute rejection include Macrophages. M1, T cells gamma delta, T cells CD4 memory activated, eosinophils, etc.

**Conclusion:** We have identified a total of 32 differential expressed transcripts and based on that, a predictive model with five significant transcripts was established, which was suggested as a highly recommended tool for the prediction of BPAR after kidney transplantation. However, an extensive study should be performed for the evaluation of the predictive model and mechanism involved.

## Introduction

With the significant improvement of quality of life for patients with end-stage renal disease, Kidney transplantation has been recognized as one of the most effective ways to treat end-stage renal disease (Garcia et al., 2012). However, various complications, such as acute rejection (AR), chronic allograft dysfunction, and immunosuppressive-related nephrotoxicity, still severely limit its wide application and endanger the outcome of allografts and recipients (Joseph et al., 2001). In recent years, with the application of various immunosuppressive drugs, the incidence of rejection after kidney transplantation has dropped dramatically (Wu et al., 2009). However, due to the occurrence of rejection mediated by various immune cells and antibodies, the function loss of the transplanted kidney is still the main problem, accounting for approximately 30% of the renal allograft loss, as well as the increased risk of chronic allograft dysfunction, and poor long-term results. Kidney transplant biopsy, an invasive procedure, is currently considered to be the gold standard for diagnosis of rejection. In order to diagnose AR, clinicians would refer to clinical tests, such as serum creatinine, the elevation of proteinuria (Solez et al., 2008; Singh et al., 2019). Therefore, it is important to understand the mechanism of AR and early prediction models.

In recent years, with the application and development of gene sequencing technology and large-scale data analysis technology in genetic diagnosis and analysis, a growing number of differential genes have been used for disease prediction and diagnosis. With the widespread application of next-generation sequencing technology, a deeper understanding of rejection reactions was observed after kidney transplantation. From the previous appearance to the cellular level, more attention was gained for gene expression. The impact of the differences in the genetic level to explain the mechanism of various rejection reactions has become the current mainstream (Sadowski et al., 2015; Chen et al., 2018; Haas et al., 2018). Rejection after renal transplantation includes three main types of allograft rejection: hyperacute rejection that occurs a few minutes after transplantation, AR that occurs a few days to a few months after transplantation, and chronic allograft rejection that occurs long after transplantation. Rejection. AR is further divided into antibody-mediated rejection (ABMR), T cell-mediated rejection (TCMR), C4d negative ABMR and mixed rejection, etc (Dorr et al., 2018).

In this study, we retrieved data sets related to AR after kidney transplantation from the Gene Expression Omnibus (GEO) database, including (GSE129166, GSE48581, GSE36059, and GSE98320). We set up the first three data sets as the validation set and the fourth data set as the training set. R language was used to analyze differential genes, combined with immune cell infiltration and pathway enrichment analysis, established a biopsy-proven acute rejection (BPAR) prediction model, and explained its signal pathway.

## Materials and Methods

### Data Retrieval and Organization

The whole process is shown in Supplemental Figure S1. We searched the dataset with “Kidney transplant and Acute rejection” as the search term from GEO official website. Finally, GSE129166, GSE48581, GSE36059, and GSE98320 were selected as research objects by us after being screened (Halloran et al., 2013; Reeve et al., 2013; Reeve et al., 2017; Van Loon et al., 2019). The samples in the four data sets are all kidney biopsy specimens. The data processing process includes original data download, probe annotation, missing value completion, and *p* difference removal. This process is jointly completed by two professional bioinformatics analysts. The samples in each data set are divided into BPAR and No-BPAR groups respectively, and the detailed information of the grouping is shown in Table 1.

**TABLE 1:** GEO dates.

GEO no	Platform	Species	Tissues	No-BPAR	BPAR	Total	Group
GSE129166	GPL570	Homo sapiens	kidney biopsy	160	52	212	validation set
GSE48581	GPL570	Homo sapiens	kidney biopsy	222	84	306	
GSE36059	GPL570	Homo sapiens	kidney biopsy	281	130	411	
GSE98320	GPL15207	Homo sapiens	kidney biopsy	774	434	1,208	training set
GSE129166	Gene expression profiling in patients with a kidney transplantation						
GSE48581	Potential impact of microarray diagnosis of T cell-mediated rejection in kidney transplants: the INTERCOM study						
GSE36059	Molecular diagnosis of T cell-mediated rejection in human kidney transplant biopsies; Molecular diagnosis of antibody-mediated rejection in human kidney transplants						
GSE98320	Assessing rejection-related disease in kidney transplant biopsies based on archetypal analysis of molecular phenotypes						

### Differential Gene Analysis

The differential genes in the data set are extracted by us, through the “limma” package in the R language, with logFoldChange = 0.5, adjustP = 0.05 as the filter value, then take the intersection of the differential genes of these four gene sets, and find the common differential genes for the next step of the analysis. All statistical data and figures were analyzed by using R 4.0.4. Limma is an R package for the analysis of gene expression microarray data, especially the use of linear models for analyzing designed experiments and the assessment of differential expression. Limma provides the ability to analyze comparisons between many RNA targets simultaneously in arbitrary complicated designed experiments. Empirical Bayesian methods are used to provide stable results even when the number of arrays is small. The normalization and data analysis functions are for two-color spotted microarrays. The linear model and differential expression functions apply to all microarray technologies including Affymetrix and other single-channel oligonucleotide platforms (Ritchie et al., 2015; Phipson et al., 2016).

### Establishment of the Prediction Model

Based on the differential genes found in the first step, we divide the training set samples into BPAR and No-BPAR according to the puncture results of the samples and perform single-factor logistic regression model predictions in turn, and the random forest graph reduces the dimensionality to find the correlation. For stronger genes, the logistic regression prediction model is finally used to determine the final prediction model gene. In all the processes, the *p*-value is less than 0.05.

### Immune Cell Infiltration

We use the CIBERSORT algorithm to quantify 22 kinds of immune cell infiltration analysis for each sample in the training set, and then use the ggstatsplot package in the R language to draw the immune cell infiltration map (Bindea et al., 2013; Finotello and Trajanoski, 2018).

### Functional Enrichment Analysis

In order to find out the pathways of differential genes, we performed Gene Ontology (GO) analysis and Kyoto Encyclopedia of Genes and Genomes (KEGG) pathway analysis, and GSEA functional enrichment analysis on the differential genes in each data set. GO Analysis: The R package: clusterProfiler, was applied. OrgDb = org. Hs.eg.db, pvalueCutoff = 0.05, qvalueCutoff = 0.05, KEGG enrichment analysis, organism = “hsa”, pvalueCutoff = 0.05, qvalueCutoff = 0.05, GSEA analysis application GSEA 4.1.0, set up conditions (number of permutation:1,000; permutation type:phenotype; enrichment statistics; weighted; metric for ranking genes:signal2noise; gene list sorting mode:real; gene list ordering mode; descending; max size; exclude larger sets:500; min size:exclude smaller sets:500; plot graphs for the top sets of each phenotype:20). In addition, in order to explore the interaction of differential genes in the acute rejection of kidney transplantation, we applied protein interaction network analysis to illustrate the possible associations between differential genes through the STRING website.

## Results

### Detection of Differential Expressed Genes

**FIGURE 1:**
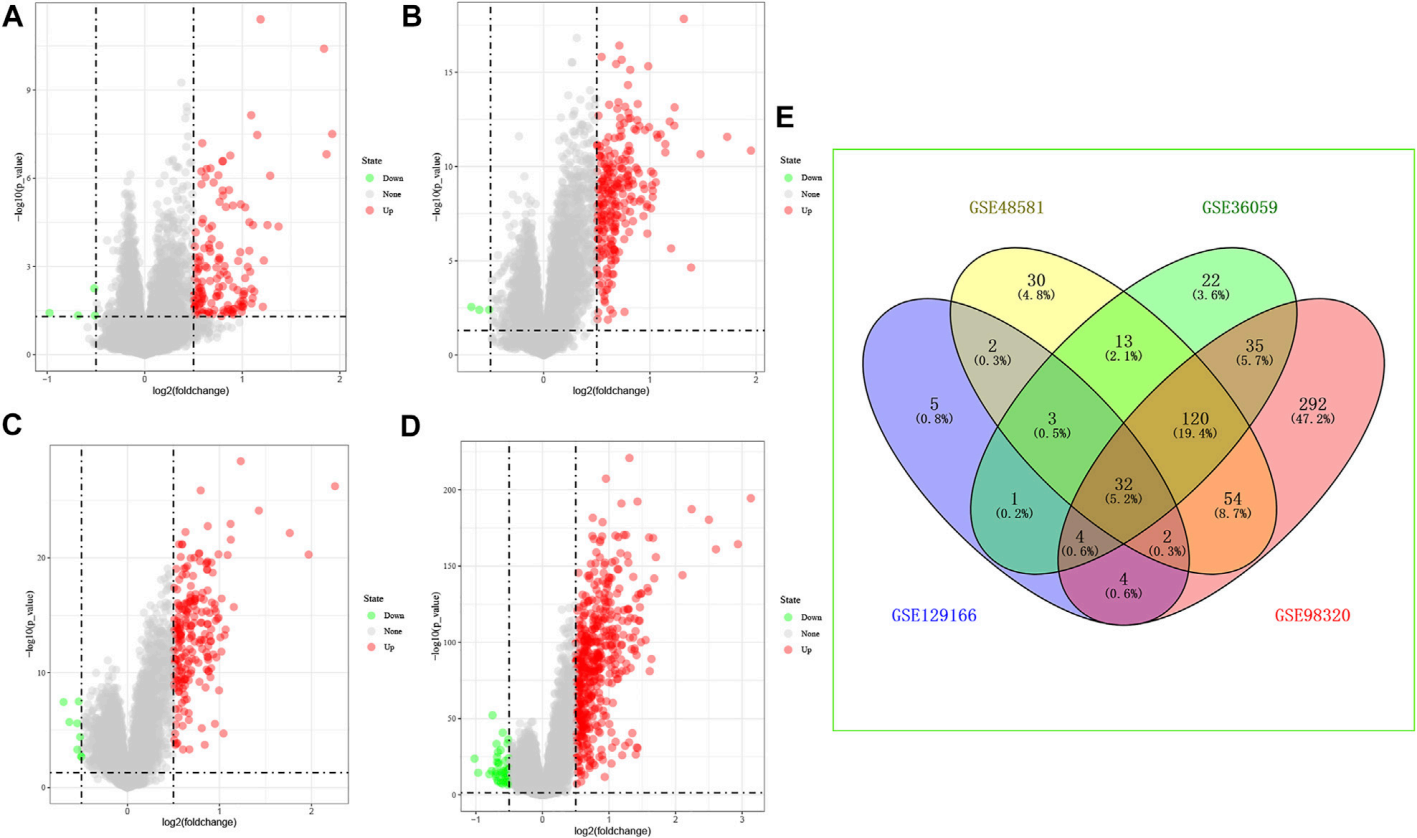
Differential gene expression, **(A)**: GSE129166 **(B)**: GSE48581 **(C)**: GSE36059 **(D)**: GSE98320 **(E)**: Common differential gene expression of the four data sets. **(A–D)**: Red represents up-regulated differential genes, green represents down-regulated differential genes.

We have reviewed a total of four datasets in the GEO database, and four studies with BPAR and its control group were included in our study for further analysis, Then, 32 common differential genes were obtained in the pooled analysis, the information and expression of which were shown in Figures 1A–E.

### Establishment of Predictive Model

**FIGURE 2:**
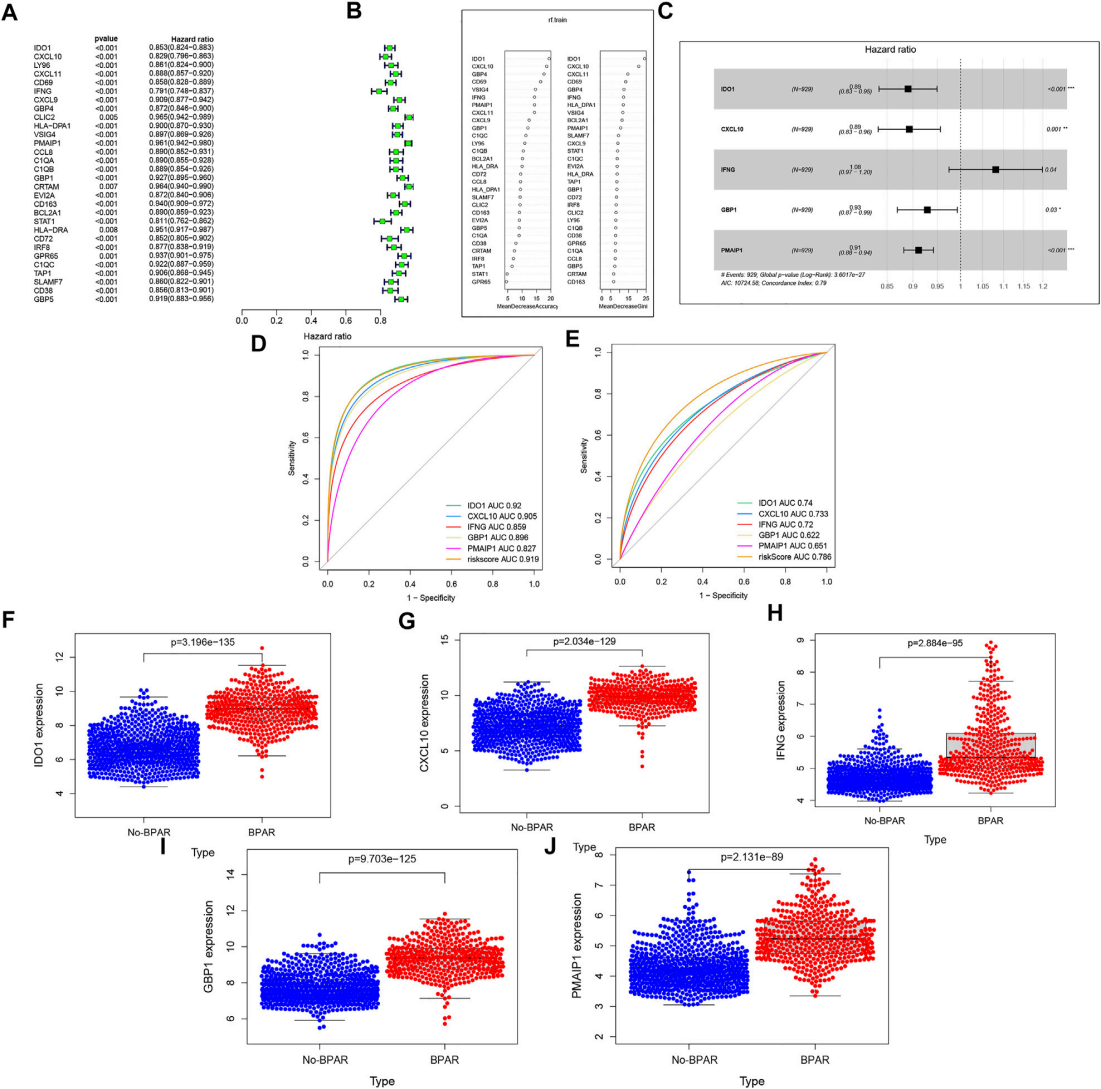
**(A)**: the single-factor prediction model of the training set. **(B)**: According to the expression matrix of these 30 genes in the training set, the result of random forest dimensionality reduction. **(C)**: the multi-factor prediction model of the expression matrix of these 10 genes in the training set. **(D)**: ROC curve of the prediction result of the training set. **(E)**: ROC curve of the prediction result of the validation set **(F–J)**: the expression levels of these five target genes in the samples. Blue represents the expression in the No-BPAR group and orange represents the expression in the BPAR group with a *p*-value less than 0.05.

To establish the predictive model, the datasets (GSE98320) was identified as the training set and 30 target genes were obtained. The result is shown in Figures 2A, Next, random forest dimensionality reduction was performed and 30 target genes were arranged in descending order according to their correlation with BPAR (Figures 2B). To reduce dimensionality through random forest modeling, the first 10 target genes were included to construct a multi-factor model for the training set, includingGBP1, CXCL9, CXCL11, PMAIP1, IFNG, VSIG4, CD69, GBP4, CXCL10, IDO1 (Figures 2C) (Mosey and Mitchell, 2020). During the process, the prediction results of the last five target genes (IDO1, CXCL10, IFNG, GBP1, GBP1) were obtained with regard (Figures 2D). These five prediction models have a good diagnostic effect on BPAR The AUC value for the comprehensive diagnosis of the five target genes was 0.919, and the 95% CI was 0.902–0.939. Furthermore, the area under the curve (AUC) value and 95% CIs of each gene alone and the comprehensive diagnosis are calculated (IDO1: AUC = 0.92.95% CIs, 0.902–0.939; CXCL10: AUC = 0.905,95% CIs, 0.886–0.927; IFNG: AUC = 0.859,95% CIs, 0.837–0.881; GBP1: AUC = 0.896,95% CIs, 0.875–0.917; PMAIP1: AUC = 0.827,95% CIs, 0.804–0.853. The AUC value for the comprehensive diagnosis of the five target genes was 0.919, and the 95% CI was 0.902–0.939. The predictive diagnosis results of these five target genes for BPAR in the verification set are shown in Figures 2E. The area under the curve (AUC) value and 95% CI of each gene alone and the comprehensive diagnosis is calculated. IDO1: AUC = 0.74,95% CIs, 0.705–0.774; CXCL10: AUC = 0.733,95% CIs, 0.697–0.768; IFNG: AUC = 0.72,95% CIs, 0.682–0.754; GBP1: AUC = 0.622,95% CIs, 0.581–0.658; PMAIP1: AUC = 0.651,95% CIs, 0.617–0.692. The AUC value for the comprehensive diagnosis of the five target genes was 0.786, and the 95% CI was 0.754–0.817.

We validated the data from each of the three datasets in the validation set and obtained good results, which are shown in Supplemental Figure S3A–C. In addition, we also performed precision/recall analysis on the training and validation sets, and the results are shown in Supplemental Figure S3D–E.

We extracted the ABMR and TCMR from the training set validation set separately for modeling analysis, and also obtained good results, which are shown in Supplemental Figure S4A–D.

Finally, expression levels of these five target genes in the samples were extracted, and it was found that the expression levels of these five genes in the samples of the BPAR group were significantly higher than those of the No-BPAR group (Figures2F–J).

### Functional Enrichment Analysis

We performed GO/KEGG/GESA functional enrichment analysis on the 32 differential genes common to these four data sets, and obtained the main functional enrichment pathways of these differential genes.

**FIGURE 3:**
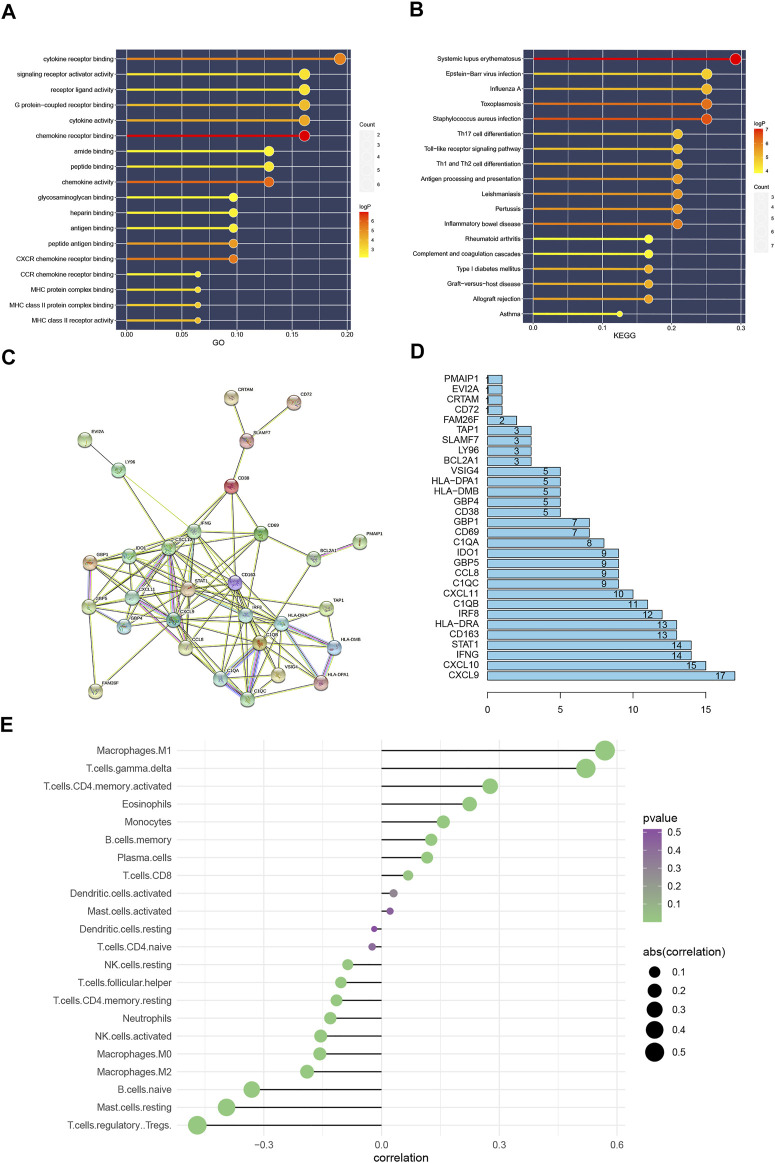
**(A)**: GO function enrichment analysis results. **(B)**: KEGG function enrichment analysis results. The color of the bar graph represents the *p* value, The color change from light to dark means that the *p* value becomes larger gradually, and the size of the endpoints represents the number of genes enriched in the pathway, the larger the endpoints the greater the number of enriched genes. **(C)**: Protein interaction network, Protein interaction network results. **(D)**: Statistics of the number of protein interactions, **(E)**: The result of immune cell infiltration. The value of the abscissa represents the correlation between the infiltration of immune cells and the occurrence of BPAR. The color change from green to purple represents the gradual increase of *p* value, and the size of the bar graph represents the size of correlation with BPAR. Correlation >0 represents immune cells positively correlated with BPAR, and correlation <0 and immune cells negatively correlated with BPAR.

Firstly, through GO functional enrichment analysis, It is found that these differential genes are mainly enriched in the following pathways (chemokine receptor binding, chemokine activity, CXCR chemokine receptor binding, cytokine receptor binding, peptide antigen binding, cytokine activity, G protein-coupled receptor binding, MHC class II receptor activity, MHC class II protein complex binding, MHC protein complex binding (Figures 3A). Next, KEGG enrichment analysis was also performed on these differential genes, and the following pathways were observed to be mainly enriched: Systemic lupus erythematosus, *Staphylococcus aureus* infection, Toxoplasmosis, Inflammatory bowel disease, Pertussis, Leishmaniasis, Antigen processing and presentation, Allograft rejection, Th1, and Th2 cell differentiation, Graft-versus-host disease, Type I diabetes mellitus (Figures 3B).

After setting the filtering conditions and filtering to a single node, protein interaction network based on the 32 DEGs was detected and a protein interaction network diagram of 30 nodes and 110 relationship pairs is obtained (Figures 3C). Figures 3D showed the number of interactions between each differential gene.

### Immune Cell Infiltration

For immune cell infiltration in BPAR, CIBERSORT algorithm was used. Combined with the risk value of our previous prediction model, four types of immune cells, including M1 Macrophages, gamma. delta T cells activated CD4 memory T cells, and eosinophils, were observed to be positively involved in the pathogenesis of BPAR (Figures 3E). Also, four types of immune cells, including T cells. Regulatory Tregs, Mast. cells. resting, B. cells.naive, Macrophages.M2, The infiltration of these types of immune cells shows a negative correlation with acute rejection.

### Gene Set Enrichment Analysis

Using GSEA software to perform KEGG function enrichment analysis on the training set, it is observed that its functions are mainly enriched in the following pathways: T cell receptor signaling pathway, natural killer cell-mediated cytotoxicity, B cell receptor signaling pathway, and endocytosis (Supplemental Figure S2). Detailed information of these pathways is shown in Table 2.

**TABLE 2:** Top 20 results of GSEA enrichment analysis.

Name	Size	ES	NES	NOM p-val	FDR q-val
KEGG_ENDOCYTOSIS	177	0.494568	2.070416	0	0.018462
KEGG_APOPTOSIS	87	0.664266	2.019174	0	0.018245
KEGG_RIG_I_LIKE_RECEPTOR_SIGNALING_PATHWAY	68	0.656445	1.995741	0	0.01618
KEGG_PROTEASOME	42	0.704777	1.991339	0	0.012468
KEGG_SNARE_INTERACTIONS_IN_VESICULAR_TRANSPORT	36	0.5059	1.969003	0.005929	0.011168
KEGG_PANCREATIC_CANCER	68	0.58511	1.958309	0	0.010169
KEGG_ANTIGEN_PROCESSING_AND_PRESENTATION	78	0.845453	1.924868	0	0.011828
KEGG_JAK_STAT_SIGNALING_PATHWAY	149	0.620666	1.901952	0	0.013328
KEGG_FC_EPSILON_RI_SIGNALING_PATHWAY	77	0.654672	1.892084	0	0.013639
KEGG_FC_GAMMA_R_MEDIATED_PHAGOCYTOSIS	92	0.686529	1.89104	0	0.012276
KEGG_CELL_ADHESION_MOLECULES_CAMS	126	0.764052	1.874987	0	0.013555
KEGG_CYTOSOLIC_DNA_SENSING_PATHWAY	50	0.737671	1.867418	0	0.013108
KEGG_SPLICEOSOME	123	0.527031	1.859101	0.010225	0.012661
KEGG_ACUTE_MYELOID_LEUKEMIA	55	0.656888	1.858955	0	0.011922
KEGG_NON_SMALL_CELL_LUNG_CANCER	53	0.533476	1.856737	0	0.011426
KEGG_T_CELL_RECEPTOR_SIGNALING_PATHWAY	106	0.753891	1.856298	0	0.010712
KEGG_TOLL_LIKE_RECEPTOR_SIGNALING_PATHWAY	98	0.780624	1.855025	0	0.010161
KEGG_NATURAL_KILLER_CELL_MEDIATED_CYTOTOXICITY	128	0.793385	1.853225	0	0.009596
KEGG_B_CELL_RECEPTOR_SIGNALING_PATHWAY	75	0.762761	1.847685	0	0.009456
KEGG_CHRONIC_MYELOID_LEUKEMIA	71	0.537865	1.801105	0.00207	0.015214

**FIGURE 4:**
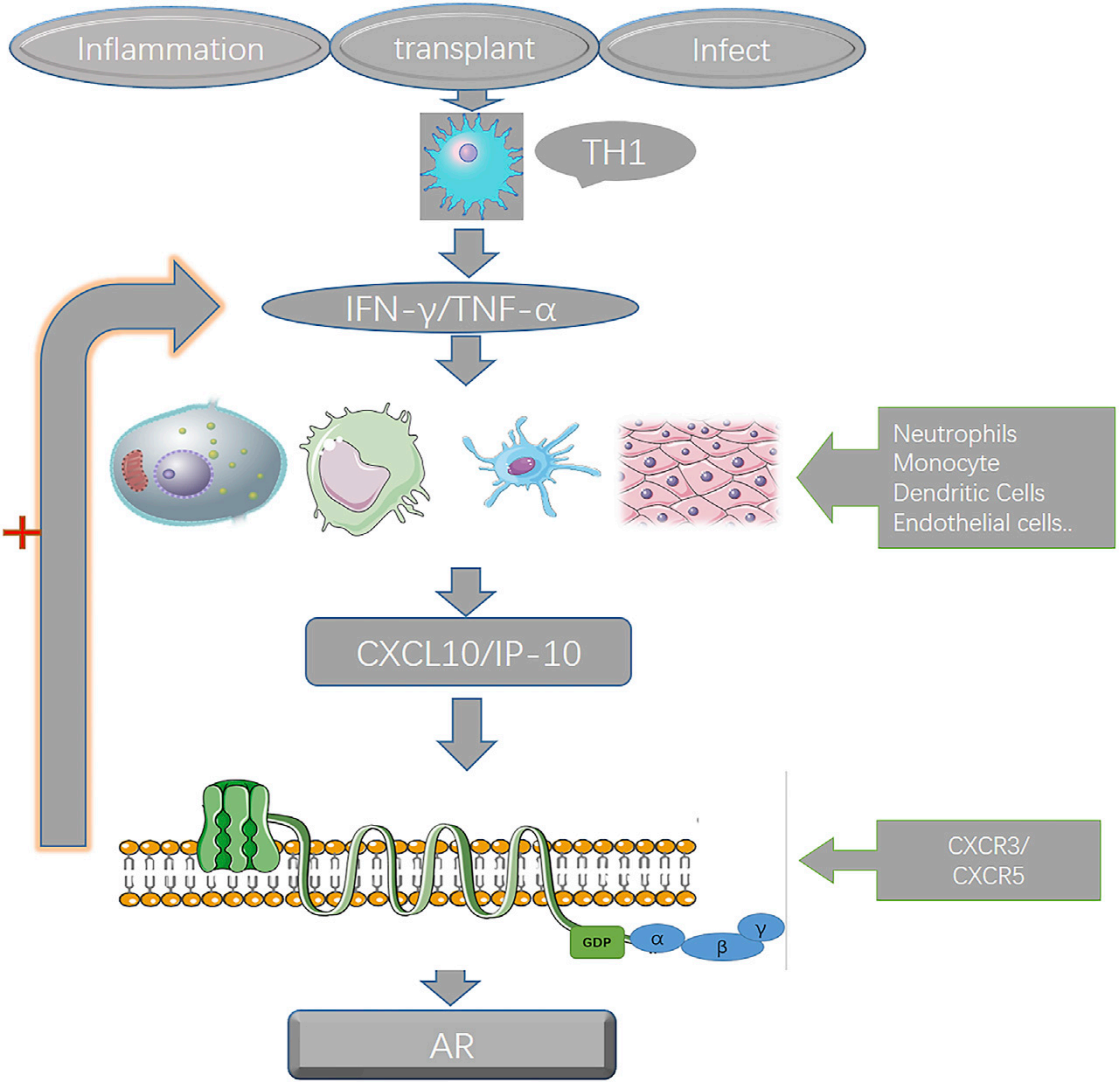
the interaction of the genes in the prediction model. After kidney transplantation, early inflammation recruits various immune cells, and the secreted CXCL10 combines with CXCR3 on various immune cells to induce the expression and secretion of IFN-*γ*, which in turn recruits more immune cells to gather. Then the inflammatory storm was aggravated, and there was a positive feedback effect of CXCL10 and IFN-*γ* during the whole process.

By consulting the literature and combining the results of the previous analysis, we hypothesized the potential interaction and mechanism of the five genes in the prediction model during the pathogenesis of BPAR (Figure 4).

## Discussion

Kidney transplantation is considered to be the most effective treatment for end-stage renal disease (Garcia et al., 2012). However, Various rejections after kidney transplantation are a major problem (Rodrigo et al., 2020). In this study, we first applied the Limma R package and the related analysis to analyze the target data set in the GEO database, analyzed the differential genes of the puncture samples, found the differential genes, and then established the BPAR prediction model. This prediction The model includes five genes (IDO1, CXCL10, IFNG, GBP1, PMAIP1). Specific information is shown in Table 3. These prediction models have good prediction results for BPAR. In addition, our analysis of differential genes also explains the possible mechanism of BPAR after kidney transplantation, which provides a reference value for further research.

**TABLE 3:** Introduction to the five target genes.

Id	Describe	The main function
IDO1	Indoleamine 2,3-dioxygenase 1	Rate-limiting enzyme of tryptophan catabolism
CXCL10	C-X-C motif chemokine 10	Recruit immune cells
IFNG	Interferon gamma gene	Inflammatory factors
GBP1	Guanylate-binding proteins	cell signaling pathway coupling protein
PMAIP1	phorbol ester-12-myristate-13-acetate inducible protein 1	Apoptotic protein

Indoleamine 2,3-dioxygenase 1 (IDO1) is a rate-limiting enzyme that can degrade tryptophan through the kynurenine pathway (Uyttenhove et al., 2003). Ischemia-reperfusion injury is an important cause of renal inflammatory response after kidney transplantation. During the process of ischemia-reperfusion, the concentration of IDO1 in the kidney tissue increases significantly, which may be an enabler of the inflammatory response after kidney transplantation, which is the basis for subsequent immune cell infiltration and a series of inflammatory storms. How to reduce the concentration of IDO1 has become a therapeutic direction to reduce inflammation, as described in the article by Eleftheriadis, T (Eleftheriadis et al., 2021). In a steady state, IDO1 expression is limited to endothelial cells in the placenta and lungs, mature dendritic cells in secondary lymphoid organs, and epithelial cells scattered in the female reproductive tract. (Théate et al., 2015). However, under inflammatory conditions, interferon gamma strongly induces the expression of IDO1 (Dai and Gupta, 1990). Many studies have shown that the increase in IDO1 is related to the occurrence of cancer and rejection after organ transplantation (Iversen et al., 2015; Bilir and Sarisozen, 2017; Hornyák et al., 2018). In the early stage after transplantation, the inflammatory response induced by various immune cells is the main reason for the impaired function of the graft. Dendritic cells play an important role in acute rejection as early antigen-promoting cells. Dendritic cells (DC) Induce the production of early inflammatory mediators through typical NF-*β* signals, the expression of indole-2,3-dioxygenase (IDO) (Tas et al., 2007). IFN-*γ* is one of the most powerful inducers of IDO in human DCs (Heitger, 2011). C-X-C motif chemokine 10 (CXCL10) is a small cytokine that belongs to the CXC chemokine family. CXCL10 is also known as interferon (IFN)-γ-inducible protein 10 (IP-10) (Lazzeri and Romagnani, 2005), CXCL10 participates in various immune responses such as various autoimmune diseases of the human body, tumor immunity, organ transplant rejection, etc (Antonelli et al., 2014). CXCL10 is an effective chemotactic agent for a variety of immune cells, such as activated type 1 T helper cells (Th1), natural killer cells (NK), dendritic cells (DC), *γδ* T cells, and macrophages (Bonecchi et al., 1998; Romagnani and Crescioli, 2012). It is secreted by a variety of cell types, including immune cells (leukocytes, neutrophils, eosinophils, and monocytes) and non-immune cells (epithelial cells, endothelial cells, keratinocytes, and stromal cells). The increase in CXCL10 production in the circulating blood of the transplant or organ recipient is related to the increase in the concentration of CXCL10 in the biological fluids (both serum and plasma) (Romagnani et al., 2001; Romagnani and Crescioli, 2012). CXCL10, like CXCL11 and CXCL9, has an IFN-*γ* induction function and exerts a biological effect by binding to CXCR3 of the seven transmembrane G protein-coupled receptor (GPCR) (Kouroumalis et al., 2005), CXCL10 has two secretion methods: paracrine and autocrine (Lo et al., 2010). CXCR3 is expressed on the surface of a variety of immune cells, including activated T cells and NK cells, DC, macrophages, and B cells. CXCR3 may promote the movement of immune cells in target tissues (Loetscher et al., 1998). After kidney transplantation, early inflammation recruits various immune cells, and the secreted CXCL10 combines with CXCR3 on various immune cells to induce the expression and secretion of IFN-*γ*, which in turn recruits more immune cells to gather. Then the inflammatory storm was aggravated, and there was a positive feedback effect of CXCL10 and IFN-*γ* during the whole process. The specific mechanism of action is shown in Figure 4. The concentration of CXCL10 in urine is related to the severity of immune inflammatory response, and it has a good role in detecting rejection after transplantation, especially the predictive role of TCMR is worth studying (Hu et al., 2004; Raza et al., 2017; Blydt-Hansen et al., 2021). Interferon gamma gene (IFNG), as a classical immune response and inflammatory response cytokine, also plays a great role in the rejection of organ transplantation, in the induction of Treg cells, and the immunity mediated by Tregs that produce IFNG plays an important role in inhibition (Lazzeri et al., 2002; Daniel et al., 2014). Guanylate-binding proteins (GBPs) family is an important cell signaling pathway coupling protein, which plays an important role in cell signal transduction, immune cell apoptosis, inflammatory cell infiltration, and bacterial virus infection rejection (Fisch et al., 2019). GBP1 is involved in the regulation of cell membranes, cytoskeleton and cell cycle processes. The expression of GBP1 is strongly stimulated by inflammatory factors such as interferon-gamma (IFN- gamma) and inhibits cell proliferation in an inflammatory environment (Honkala et al., 2019). This also happens to be manifested in the strong inflammatory response after kidney transplantation. Previous studies have also used GBP1 as a predictive model for acute rejection after renal transplantation, and it has a good predictive effect (Van Loon et al., 2019). PMAIP1 (phorbol ester-12-myristate-13-acetate inducible protein 1), also known as noxa (meaning injury in Latin), or APR (immediate early response protein), is a family of Bcl-2 proteins A member of the pro-apoptotic group (Janus et al., 2020). Pmaip1 is a p53-responsive gene, which encodes a protein that causes p53-dependent apoptosis caused by DNA damage (Sodja et al., 1998).

Then we analyzed the immune cell infiltration in the sample that we studied, through the CiberSort algorithm that is widely used by everyone. (Bindea et al., 2013; Finotello and Trajanoski, 2018). This algorithm compares the gene expression matrix with the standard immune cell infiltration gene expression matrix to obtain the immune cell infiltration spectrum of the sample. The results also confirmed the types of immune cells that are positively related to the occurrence of BPAR, which provides a direction for the following research. From our results, it can be seen that M1 macrophages have the strongest correlation with rejection. M1 macrophages may cause damage to the microvessels and renal tubular epithelial cells in the transplanted kidney through the action of TNF-α/IFN and other cytokines, The successive amplification of the inflammatory response recruits more T cell aggregation, forming a positive feedback effect, and further leading to the occurrence of acute rejection. And in the whole process, it is unscientific to use a single cell to explain the occurrence of acute rejection. It is the joint action of multiple immune cells, such as (Macrophages.M1, T. cells.gamma.delta, T. cells.CD4. memory.activated, Eosinophils), which all play an important role in this process.

In order to further study the mechanism of our prediction model, we performed a functional enrichment analysis of these differential genes, the enrichment results of GO/KEGG show that the enrichment functions of the differential genes we obtained are mainly concentrated in cell signal transduction, immune cell recruitment, and cytokine receptors. This also happens to be combined with our previous work to further explain the role of immune cell infiltration in rejection after renal transplantation., By consulting the literature, it is found that these signaling pathways play an important role in the immune response after organ transplantation. In particular, the four pathways obtained in the GSEA enrichment analysis have attracted our attention., (KEGG_T_CELL_RECEPTOR_SIGNALING_PATHWAY, KEGG_NATURAL_KILLER_CELL_MEDIATED_CYTOTOXICITY, KEGG_B_CELL_RECEPTOR_SIGNALING_PATHWAY, KEGG_ENDOCYTOSIS). More importantly, the differential genes in our prediction model play an important role in these pathways.

However, there are still shortcomings in our entire research process, First of all, the description of the mechanism of action described in this article is not specific enough. We just aggregate the causes of acute rejection after kidney transplantation into several immune cells and several signal pathways through the analysis of differential genes. The description of the mechanism is not specific enough. Secondly, the description of the signal pathway is derived from previous studies by others and lacks our verification process.

## Conclusion

In summary, we analyzed the gene expression matrix of BPAR samples in the GEO database, obtained differential genes, established a prediction model for transplanted kidney BPAR (IDO1, CXCL10, IFNG, GBP1, PMAIP1), and passed the immune cell infiltration analysis to obtain the related acute rejection Immune cells (Macrophages.M1, T. cells. gamma. delta, T. cells.CD4. memory.activated, eosinophils), next GO/KEGG/GSEA and other functional enrichment analysis, further analyze the mechanism of differential genes in the prediction model, and provide a reference value for further research.

## Data Availability

The original contributions presented in the study are included in the article/Supplementary Material, further inquiries can be directed to the corresponding authors.

## Abbreviations

ABMR, antibody-mediated rejection (ABMR); AR, acute rejection; BPAR, biopsy-proven acute rejection; GEO, Gene expression omnibus database; GO, Gene Ontology analysis; GSEA, Gene Set Enrichment Analysis; KEGG, Kyoto Encyclopedia of Genes and Genomes (KEGG) pathway analysis; TCMR, T cell-mediated rejection (TCMR).
