# Supplementary figures and images for: Biological Characteristics and Predictive Model of Biopsy-Proven Acute Rejection (BPAR) After Kidney Transplantation: Evidences of Multi-Omics Analysis
Source: Front Genet. 2022 Mar 21;13:844709. doi: 10.3389/fgene.2022.844709 (PMC9037533; doi:10.3389/fgene.2022.844709)

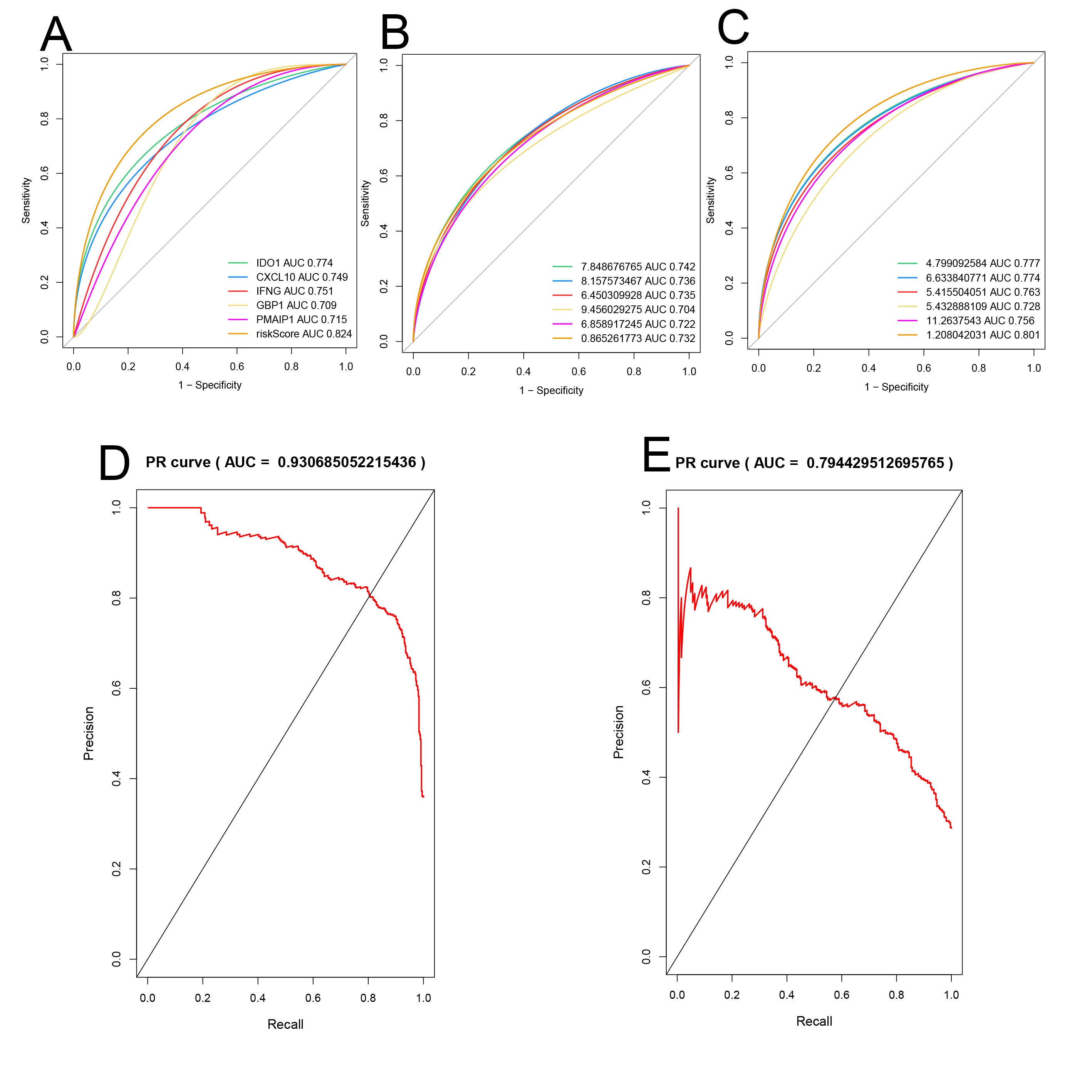

Supplement: Supplementary file 1 [file Image3.TIF]

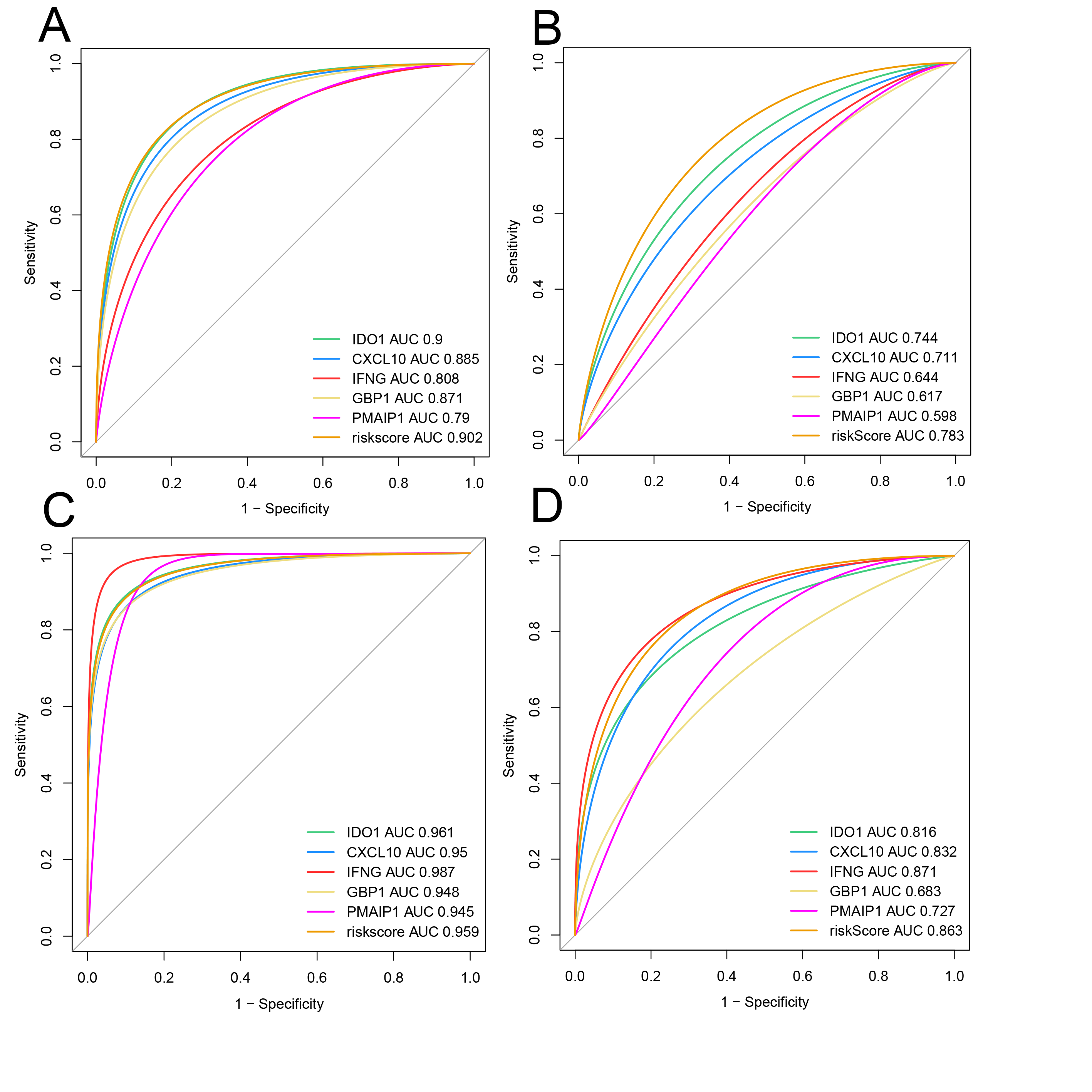

Supplement: Supplementary file 2 [file Image4.TIF]

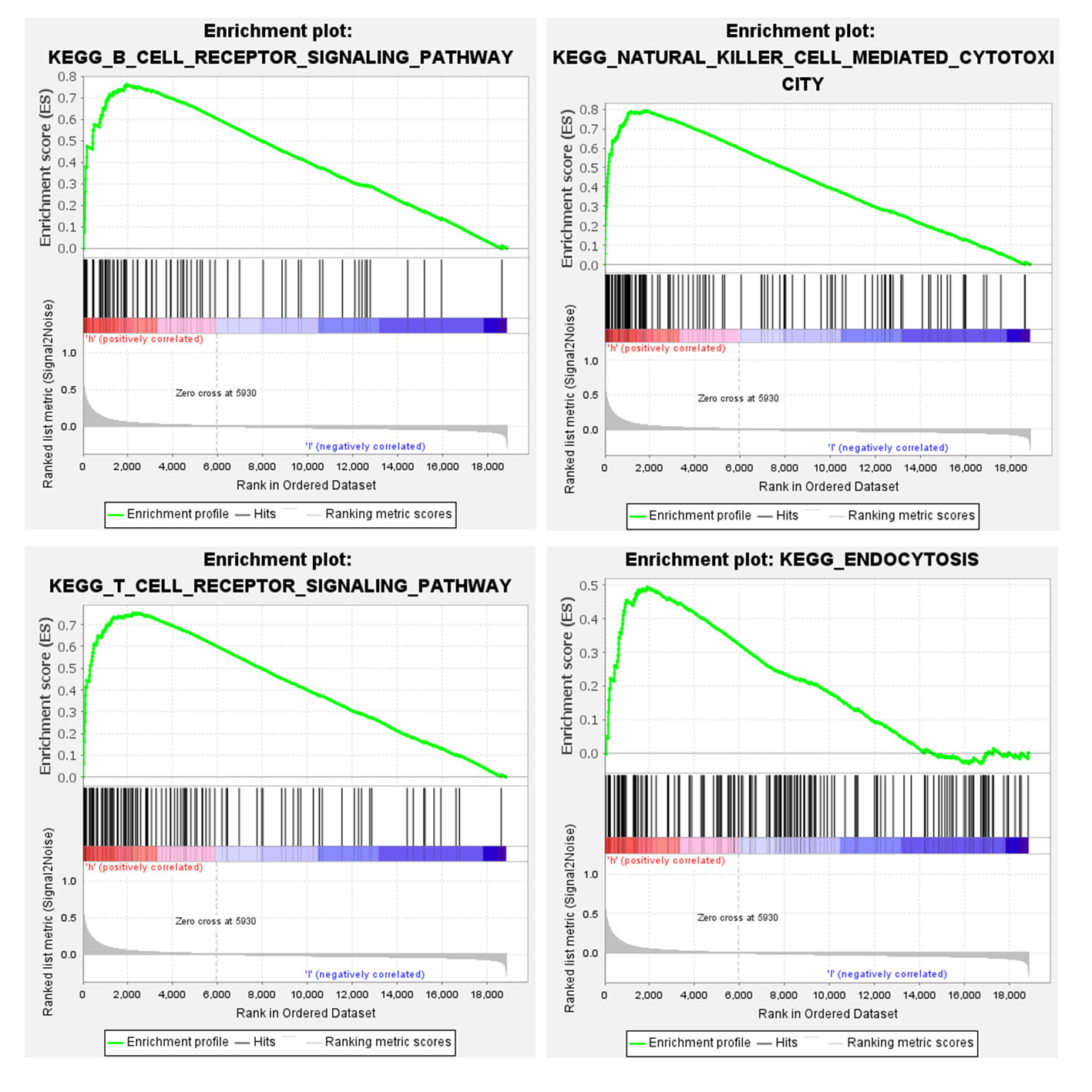

Supplement: Supplementary file 3 [file Image2.TIF]

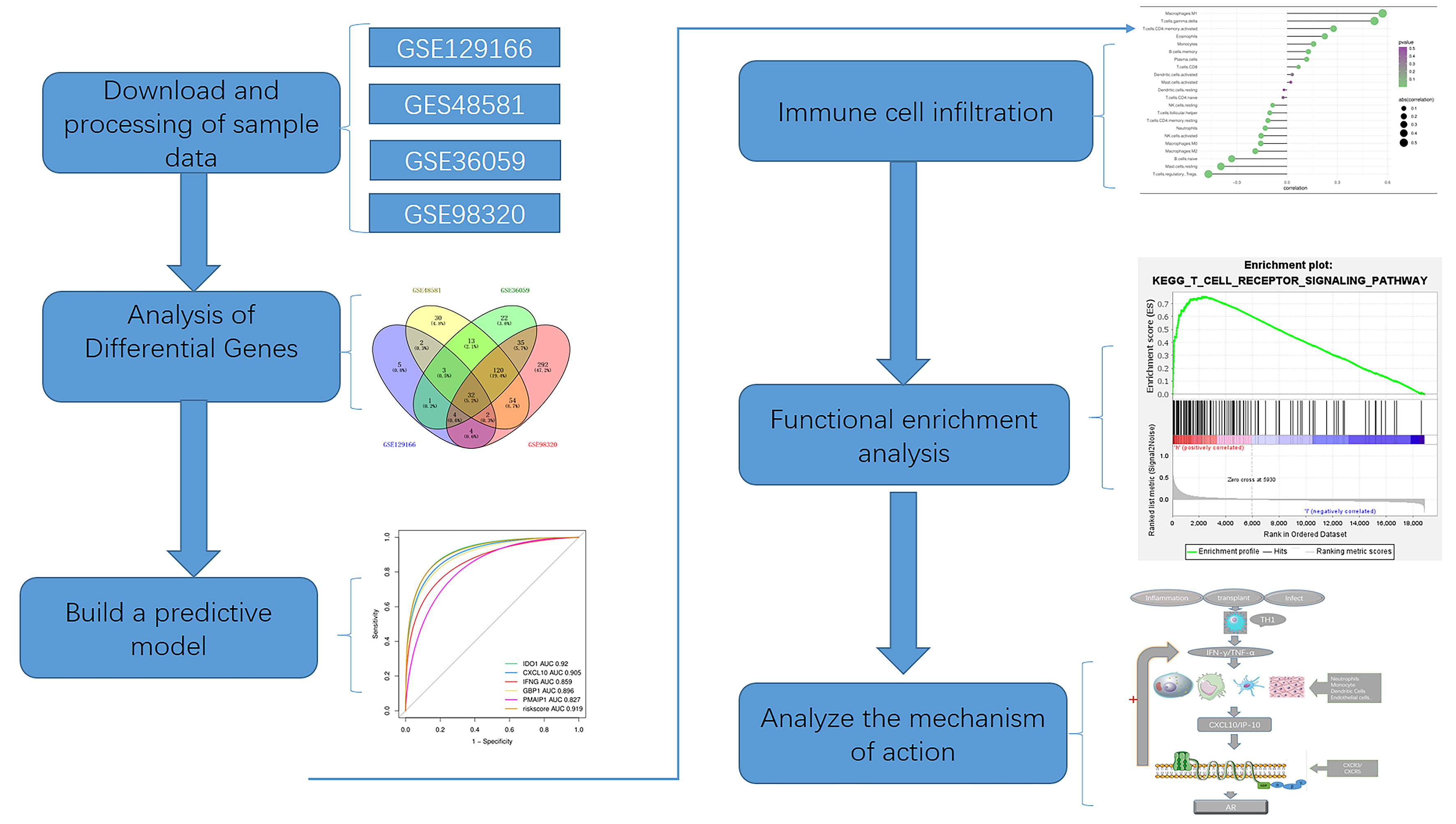

Supplement: Supplementary file 4 [file Image1.TIF]
